# Primaquine as a Candidate for HHV-8-Associated Primary Effusion Lymphoma and Kaposi’s Sarcoma Treatment

**DOI:** 10.3390/cancers14030543

**Published:** 2022-01-21

**Authors:** Adélie Gothland, Valentin Leducq, Philippe Grange, Ousmane Faye, Laurianne Beauvais Remigereau, Sophie Sayon, Nathalie Désiré, Aude Jary, Emmanuel Laplantine, Almoustapha Issiaka Maiga, Nicolas Dupin, Anne-Geneviève Marcelin, Vincent Calvez

**Affiliations:** 1 Service de Virologie, Hôpital Pitié Salpêtrière, Institut Pierre Louis d’Epidémiologie et de Santé Publique (iPLESP), INSERM UMR_1136, Sorbonne Université, 75013 Paris, France; valentin.leducq@sorbonne-universite.fr (V.L.); lauriannebr@lbrconseil.fr (L.B.R.); sophie.sayon@aphp.fr (S.S.); aude.jary@aphp.fr (A.J.); anne-genevieve.marcelin@aphp.fr (A.-G.M.); vincent.calvez@aphp.fr (V.C.); 2 Cutaneous Biology Lab, INSERM U1016, UMR8104, Institut Cochin, Université de Paris, 24 Rue du Faubourg St Jacques, 75014 Paris, France; philippe.grange@aphp.fr (P.G.); nicolas.dupin@aphp.fr (N.D.); 3 Service de Dermatologie, CeGGID et CNR IST Bactériennes, Hôpital Cochin Site Port Royale, AP-HP, Groupe Hospitalier Paris Centre Cochin-Hôtel Dieu-Broca, 123 Boulevard de Port Royal, 75014 Paris, France; 4 Département de Dermatologie, Faculté de Médecine et de Pharmacie, Université de Bamako, Bamako BP 1805, Mali; faye_o@yahoo.fr; 5 Institut Pierre Louis d’Epidémiologie et de Santé Publique, INSERM, Assistance Publique—Hôpitaux de Paris, Sorbonne Université, 75012 Paris, France; nathalie.desire@sorbonne-universite.fr; 6 Center for Immunology and Microbial Infections—CIMI-Paris, Sorbonne Université, INSERM, CNRS, 75013 Paris, France; emmanuel.laplantine@upmc.fr; 7 Unité d’Epidémiologie Moléculaire de la Résistance du VIH aux ARV, SEREFO, FMOS, University of Sciences, Techniques and Technologies of Bamako, Bamako BP 1805, Mali; almoustapha@gmail.com; 8 Clinical and Microbiology Laboratory, University Hospital Gabriel Toure, Bamako BP 1805, Mali

**Keywords:** human herpesvirus 8, Kaposi’s sarcoma, primary effusion lymphoma, primaquine diphosphate, oxidative stress, apoptosis

## Abstract

**Simple Summary:**

Primaquine diphosphate is introduced as a promising therapeutic candidate for HHV-8-associated diseases by inducing specific cytotoxicity in vitro through ROS- and ER stress-mediated apoptosis. PQ presented a promising anti-tumor effect in an in vivo PEL mouse model and in KS patients within a pilot clinical study.

**Abstract:**

Human Herpesvirus 8 (HHV-8) is associated with three main severe orphan malignancies, Kaposi’s sarcoma (KS), multicentric Castleman’s disease (MCD), and primary effusion lymphoma (PEL), which present few therapeutic options. We identified the antimalarial primaquine diphosphate (PQ) as a promising therapeutic candidate for HHV-8-associated PEL and KS. Indeed, PQ strongly reduced cell viability through caspase-dependent apoptosis, specifically in HHV-8-infected PEL cells. Reactive oxygen species (ROS)- and endoplasmic reticulum (ER) stress-mediated apoptosis signaling pathways were found to be part of the in vitro cytotoxic effect of PQ. Moreover, PQ treatment had a clinically positive effect in a nonobese diabetic (NOD)/SCID xenograft PEL mouse model, showing a reduction in tumor growth and an improvement in survival. Finally, an exploratory proof-of-concept clinical trial in four patients harboring severe KS was conducted, with the main objectives to assess the efficacy, the safety, and the tolerability of PQ, and which demonstrated a positive efficacy on Kaposi’s sarcoma-related lesions and lymphedema.

## 1. Introduction

The oncogenic Kaposi’s sarcoma-associated herpesvirus (KSHV, also known as Human Herpesvirus 8, HHV-8) is a principal causative agent of several human cancers including Kaposi’s sarcoma (KS), multicentric Castleman’s disease (MCD), and primary effusion lymphoma (PEL). These HHV-8-associated diseases, all classified as orphan diseases, represent important and difficult to treat clinical problems, with very few therapeutic options. These diseases are frequently associated with human immunodeficiency virus (HIV) infection, but also with other different immune deficiency associated diseases and/or treatments. Although the success of combination antiretroviral therapy (cART) has improved the outcomes of HIV-infected patients, its benefits are very limited in patients with PEL and MCD, and KS remains the most common malignancy in HIV-infected individuals [1,2]. The use of combination cytotoxic chemotherapies represents a standard approach for PEL and systemic KS [3]. However, the toxicity of systemic chemotherapy synergizes with those caused by antiretroviral therapy or immune suppression and the availability of other efficient agents, further limiting treatment efficacy [4,5]. Effective and less toxic approaches, which target either the HHV-8 virus itself, infected cells, or key cellular pathways, are thus an unmet medical need. Given the low level of virus lytic infection in patients with PEL and KS, the expectation of success with antiviral anti-herpesvirus therapy is low [1]. Indeed, HHV-8, as like other herpesviruses, has developed many mechanisms to establish persistent latent infection, which remains predominant in most infected cells [6]. Latency is a strategy for HHV-8 to hinder the destruction of HHV-8-infected tumor cells by facilitating host immune evasion and promoting tumor cell survival and proliferation through the expression of a limited number of oncogenic latent genes [7,8]. Importantly, the HHV-8 transcription pattern and the survival mechanisms associated with viral latency are very similar in different infected cell types, from primary latent infection of endothelial cells to long-term latency of lymphoma cells [9]. Hence, the molecular signaling pathways involved in the survival of infected cells represent a major target for the eradication of HHV-8-infected cells and, ultimately, for the curing of the typical malignancies associated with HHV-8. In the present study, we identified primaquine diphosphate (PQ) as a new and very promising targeted therapeutic drug, inducing cell death through apoptosis in vitro, specifically in HHV-8-infected PEL cell lines. We showed that PQ presents clinical anti-tumor efficacy in an in vivo nonobese diabetic (NOD)/SCID PEL mouse model and in humans harboring severe KS.

## 2. Materials and Methods

### 2.1. Cells

HHV-8-infected primary effusion lymphoma B-cell lines, BC-3 (CRL-2277) and BCP-1 (CRL-2294), and HHV-8-uninfected Burkitt’s lymphoma DG-75 cell line (CRL-2625) were obtained from ATCC (Manassas, VA, USA) and grown in RPMI 1640 GlutaMAX (Gibco, Thermofischer, Waltham, MA, USA) medium with 10% fetal bovine serum (FBS; Gibco, Thermofischer, Waltham, MA, USA) and 1% amikacin/vancomycin (Mylan, PA, USA). Human Fetal Lung Fibroblast MRC-5 cells (ATCC CCL-171, Manassas, VA, USA) were cultured in Eagle’s Minimum Essential Medium (EMEM, ATCC 30-2003, Manassas, VA, USA) with 10% FBS and 1% penicillin/streptomycin (Gibco, Thermofischer, Waltham, MA, USA). Three cell types of skin were used, primary normal human epidermal keratinocyte, adult (NHEK-Ad; Lonza, Basel, Switzerland) and primary normal human dermal fibroblast, adult (NHDF-Ad; Lonza, Basel, Switzerland) grown into keratinocytes growth medium (KGM^TM^ Gold; Lonza, Basel, Switzerland) and fibroblast growth basal medium (FBM™; Lonza, Basel, Switzerland), respectively, and human keratinocyte HaCaT cell line grown into DMEM with 10% FBS and 1 mM pyruvate. HeLa (ATCC CCL-2, Manassas, VA, USA) cells were cultured in Dulbecco’s Modified Eagle’s Medium (DMEM-GlutaMAX; Gibco, Thermofischer, Waltham, MA, USA) with 10% FBS and 1% penicillin/streptomycin. Primary umbilical vein endothelial HUVEC (ATCC PCS-100-010, Manassas, VA, USA) cells were cultured in Vascular Cell Basal Medium (ATCC PCS-100-030, Manassas, VA, USA) with growth factors (rh VEGF, rh EGF, rh FGF basic, rh IGF-1), L-glutamine, heparin sulfate, hydrocortisone hemisuccinate, 2% FBS, ascorbic acid, gentamicin–amphotericin B solution, penicillin–streptomycin–amphotericin B solution, and phenol red. All the cells were cultured at 37 °C under a humidified atmosphere containing 5% CO_2_.

### 2.2. Reagents

Primaquine diphosphate (PQ), diethyldithiocarbamate (DDTC), doxorubicin hydrochloride, sulfasalazine (SASP), *N*-acetyl-L-cysteine (NAC), and thapsigargin (Tg) were purchased from Sigma-Aldrich (Saint Louis, MO, USA).

### 2.3. Cell Viability Assay

Cell viability was assessed by using the luminescent CellTiter-Glo 2.0 assay (Promega, WI, USA), performed as described by the manufacturer. Briefly, cells (10^4^/well) were incubated in a 96-well plate in the presence of the compounds in a final volume of 100 µL for 48 h. Values were normalized to the untreated cells. The half-maximal cytotoxic concentrations (CC50) were calculated using GraphPad Prism 6.0.

### 2.4. Caspases-3/-7, -8, and -9 Assays

Apoptosis in BC-3, BCP-1, and DG-75 cells was assessed by measuring the activity of the executioner caspase-3 and -7 using the luminescent Caspase-Glo 3/7 assay (Promega, WI, USA), as described by the manufacturer. The activity of caspase-8 and caspase-9 was measured in BC-3 cells using the luminescent Caspase-Glo 8 assay and the Caspase-Glo 9 assay kit (Promega, WI, USA), respectively, and performed as described by the manufacturer. Briefly, cells (10^4^/well) were incubated in a 96-well plate in the presence of PQ in a final volume of 100 µL for 24 h. The caspases’ activity in untreated cells was considered as caspase basal activity and defined as 1.0 relative light unit. Values were normalized to the untreated cells.

### 2.5. Caspase-4 Assay

Caspase-4 activity was assessed using the fluorometric caspase-4 assay kit (abcam, Cambridge, UK), according to the manufacturer’s instructions. Briefly, after treatment with PQ (10 µM) or thapsigargin (Tg, 1 µM as positive control) for 3–30 h, BC-3 cells were lysed and proteins were extracted. In total, 200 µg of total proteins from each sample was incubated with the caspase-4 substrate at 37 °C for 1 h, and caspase-4 activity was then monitored by fluorometer. The fold-increase in caspase-4 activity was determined by comparing the results of treated cells with the level of the untreated cells.

### 2.6. Phenotypic Screening Assay

Phenotypic screening with the 2781 compounds of the Prestwick Chemical Libraries (14D1305 MP02 ST05 and 02-Nat. Mp01 D1203 ST08, Prestwick Chemical, Illkirch, France) was performed by quantification of the cell viability (CellTiter-Glo 2.0 assay) and apoptosis (Caspase-Glo 3/7 assay) in BC-3 cell line, as previously described. The Prestwick compounds were tested at a final concentration of 10 µM (DMSO 1‰) in 96-well plates. Diethyldithiocarbamate (DDTC), known to induce cytotoxicity through apoptosis in a caspase-3-dependent pathway in BC-3 cells, was used as a positive control [10].

### 2.7. Measurement of Intracellular Hydrogen Peroxide (H_2_O_2_) and Reduced Glutathione (GSH) Productions

To measure H_2_O_2_ and GSH productions, BC-3 cells were treated in a 96-well plate with PQ in a final volume of 100 µL for 24 h. Cells were then washed three times in PBS and incubated with 100 µL per wells of 5 µM 2′,7′-dichlorodihydrofluorescein diacetate (H2-DCFDA for determination of H_2_O_2_) or 5 µM monochlorobimane (for determination of GSH) in PBS for 30 min at 37 °C. Fluorescent probes were purchased from Molecular Probes (Eugene, OR, USA). After three washes, fluorescence intensity was recorded every hour over a period of 6 h. At the end of the experiment, the number of BC-3 cells was evaluated by the crystal violet assay, as described below. H_2_O_2_ and GSH were assayed by spectrofluorimetry (Fusion, PerkinElmer). Levels of reactive oxygen species (ROS) and GSH were calculated in each sample as follows: H_2_O_2_/GSH rate (arbitrary units/min/10^6^ cells) = fluorescence intensity (arbitrary units) at T6h—fluorescence intensity (arbitrary units) at To/360 min/number of BC-3 cells as measured by the crystal violet assay, and were expressed as arbitrary unit (A.U.). Cell viability assays: Crystal violet staining was used to determine the number of adherent cells in 96-well plates. Briefly, after incubation with the test compound, the culture medium was discarded, and the cells were incubated with a 0.05% crystal violet solution (Sigma) for 30 min at room temperature. After washing with PBS, 100% methanol was added, and the absorbance was spectrophotometrically measured at 540 nm on an ELISA multiwell reader. The MTT (1-(4,5-dimethylthiazol-2-yl)-3,5-diphenylformazan) assay was performed to test cell viability in 96-well plates. The cells were incubated with a 0.2% MTT solution in cell culture medium for 4 h at 37 °C. The MTT solution was then discarded and DMSO added to solubilize the MTT-formazan crystals produced in living cells. After thorough mixing, the absorbance was measured at 540 nm. H_2_O_2_ and GSH levels on untreated cells (control) were considered as 1.0 and 100%, respectively. Values were normalized to H_2_O_2_ or GSH levels of the control.

### 2.8. Measurement of Extracellular HHV-8 Virus Production by Real-Time PCR

BC-3 cells (2 × 10^6^ cells in 10 mL) were treated by PQ or SASP (as a positive control) for 12–48 h or left untreated as a negative control. Cells were then centrifuged at 1500 rpm for 15 min and supernatant was filtered through a 0.45 µM pore to remove cellular debris. Virus was pelleted down by ultracentrifugation at 36,000 rpm for 3 h. Each pellet was then treated with 100 µL of DNase I (Qiagen, Hilden, Germany) to eliminate free DNA. HHV-8 virion DNA was extracted and quantified by real-time PCR using an ORF-73 (LANA) specific primer set, as previously described [11].

### 2.9. Transcriptome Profiling by RNA-Seq

The RNA-seq of the triplicate BC-3 cells untreated or treated with 10 µM PQ for 12 h was performed on a NextSeq 500 next-generation sequencer, Illumina (75 pb paired-end sequencing; depth sequencing of 22 million reads per sample). Briefly, total RNA was purified using the Rneasy Mini Kit (Qiagen, Hilden, Germany), quantified by Qubit RNA BR assay kit (Thermofischer, Waltham, MA, USA), according to the manufacturer’s instructions. cDNA libraries were prepared from poly(A)-selected RNA and subjected to RNA deep-sequencing (RNA-seq) analysis on the genotyping and sequencing core facility (iGenSeq) at the Paris Brain Institute. Cellular RNA reads were mapped onto the human genome (hg19) using the STAR program, then quantified by RSEM and normalized with the R edgeR package to obtain count per million values (cpm). The changing expression levels of cellular transcripts within PQ-treated compared to PQ-untreated BC-3 cells were also carried out by the R edgeR package. The cut-off values selected for the analysis of the data were: *p*-value threshold: 0.05; log2foldchange threshold: 1.5; count per million (CPM) threshold: 1. The reads of the viral transcripts were mapped onto the HHV-8 genome sequence (NC_009333) using the Tophat program. Quantification of the reads was carried out by featureCounts. CPM values were calculated by the DEseq2 program, and differential expression of viral genes was also performed using the DEseq2 program. To find out what biological process or molecular function the differentially expressed genes were associated with, a pathway enrichment analysis (Gene Ontology) was also conducted with Fisher test using an R package (clusterProfiler).

### 2.10. qRT-PCR

Total RNA was purified using the RNeasy Mini Kit (Qiagen, Hilden, Germany), quantified by Qubit RNA BR assay kit (Thermofischer, Waltham, MA, USA), according to the manufacturer’s instructions. cDNA was synthesized from equivalent total RNA using SuperScript^TM^ III Reverse Transcriptase (Invitrogen, Waltham, MA, USA), according to the manufacturer’s procedures. Amplification was carried out using an ABI Prism 7500 real-time PCR system utilizing PowerUp SYBR Green Master Mix reagent (Applied Biosystems, Waltham, MA, USA), and cycle threshold (Ct) values were tabulated in triplicate for each gene of interest in each experiment. “No template” (water) and RT controls were performed in every run. Primers used for the amplification of target genes are displayed in Supplementary Table S1. The data were analyzed using the ΔC_t_ method, as previously described [12]. The ratio (R) of the target gene is expressed in a treated sample versus a control (untreated sample) in comparison to a reference gene (β-actin), according to the formula:

R = (AE_target_)^Δct target (positive control-treated sample^/(AE_β-actin_) ^Δct β-actin (positive control-treated sample)^ (1)

For the calculation of R, the real-time PCR efficiencies (Amplification Efficiencies, AE) of each target gene and β-actin were calculated.

### 2.11. PEL Xenograft Murine Model

Six-week-old female NOD/SCID mice (Charles River Laboratories, Wilmington, MA, USA) were kept at the animal care facility at the Pitié-Salpétrière Hospital, Paris. All animal studies were conducted according to protocols approved by the Animal Ethics Committee Charles Darwin N° 5 of the Paris Brain Institute (ICM; APAFIS#6542-2016082515231962). Six-week-old female mice were assigned into four groups (*n* = 6) with a median weight of 20 g. The mice received intraperitoneal (i.p) injection of 10^7^ BC-3 cells from culture diluted in 500 µL PBS (engrafted mice) or 500 µL of PBS alone (unengrafted mice) on day 0. In total, 25 mg/kg PQ (PQ-treated mice) or PBS alone (vehicle-treated mice) was intraperitoneally administrated on day 1, and 3 days a week thereafter for 66 days. The dose of administrated PQ was chosen according to previous studies on PQ-treated SCID mice [13]. Body weight gain for individual mice was recorded weekly as a criterion for ascites growth and tumorigenesis. Ascites fluid from mice i.p injected with BC-3 cells were immediately collected postmortem and measured. Reduction in body weight gain and ascites volume as an indicator of anti-tumor efficacy of PQ was evaluated in the PQ-treated engrafted group compared to the untreated engrafted group. HHV-8 viral load (HHV-8 copy number/mL) was also determined by real-time PCR using an ORF-73 (LANA) specific primer set in ascites fluid. For confirmation of PEL cell expansion within the murine model, DNA was extracted from peritoneal solid samples of BC-3 cell-grafted mice, and copy number of HHV-8 and albumin was measured with real-time PCR, as previously described [11]. Mice were monitored and euthanized when tumor burden interfered with ambulation, eating, drinking, defecation, and urination and/or when weight reached 150% of initial weight. Survival was evaluated from the first day of treatment until death.

### 2.12. Proof of Concept Clinical Trial

This study was approved by the Comité d’Ethique de la FMPOS (Faculté de Médecine et Pharmacie et Odonto Stomatologie); Université des Sciences, des Techniques et des Technologies de Bamako. Decision number 2019/34/CE/FMPOS, 11 April 2019 (Prof Amadou Diallo) and amendment decision number 2019/179/CE FMPOS, 23 December 2019 (Prof Amadou Diallo). All patients received and signed informed consent. Patient eligibility: To be eligible, patients had to be older than 18 years of age, have a proven endemic Kaposi’s sarcoma on a skin biopsy, without ulcerated lesions and with at least two evaluable lesions. Patients did not receive any other treatment for Kaposi’s sarcoma before inclusion. Patients with a history of systematic visceral injury or with severe glucose-6-phosphase-dehydrogenase (G-6-PD) deficiency; ECG abnormality; those receiving potentially hemolytic drugs or drugs that depress myeloid elements of the bone marrow; those with liver dysfunction; those with renal insufficiency with creatinine clearance <40 mL/min; those HIV1 or HIV2 positive; pregnant or breastfeeding women; and patients who had been treated with chemotherapy or immunotherapy within 4 weeks prior to treatment or who had electrolyte disorders (hypokalemia, hypo, or hypercalcemia) were not included in the study. Study design and treatment: Proof of concept study assessing the efficacy and safety of primaquine diphosphate (Wellona Pharma, Surat, India). As the World Health Organization recommends the use of primaquine at 15 mg/day, or in some cases at 30 mg/day [14], the first patients (1 and 2) were treated with 15 mg of primaquine once daily (OD) for 12 weeks. Based on the efficacy and safety results of the first two patients, the protocol was amended to the use of 30 mg of primaquine per day (15 mg twice a day: BID) for patients 3 and 4 for 12 weeks. Response assessment: At inclusion, the total number of lesions and the presence of lymphedema were reported. Partial response of a KS lesion is defined as at least a 50% reduction in the tumor size and, for lymphedema, a decrease clinically validated by the investigator. Complete response is defined as the absence of detectable target lesions and lymphedema for more than four weeks. The target lesions should be at least 5 mm long, defined as the sum of the length of the two longest perpendicular diameters. The characteristics of the target lesions and photographs were registered at each clinical visit. The lesions were to be evaluated on photographs reviewed by two dermatologists. When the definitions of complete response, partial response, or progressive disease did not apply, the response was defined as stable disease. Adverse event (AE) assessment: AEs were monitored during each visit and 4 weeks after completing therapy and until AE resolution. Laboratory assays: The following blood samples were to be taken at baseline, M1, M2, M3: haematology (blood counts and platelet count) and biochemistry (sodium, potassium, total calcium, AST, ALT, creatinine).

### 2.13. Statistics

All in vitro results are expressed as mean ± standard deviation (SD) or standard error of the mean (SEM). Statistical significance for differences between treated and untreated control groups was determined using the Welch’s *t*-test for Figure 2A and Wilcoxon Mann Whitney’s test for Figure 2B. Statistical significance for differences between treated and untreated control groups was determined using the Welch’s *t*-test for Figure 3A,B. Welch’s *t*-test was performed between NAC and vehicle groups for Figure 3C,D. Statistical significance for differences between treated and untreated control groups was determined using Wilcoxon Mann Whitney’s test for Figure 4. The data of Figures 5B, 6B and 7B were analyzed by analysis of variance (ANOVA) and Dunnett’s or Tukey’s multiple comparisons tests. Welch’s *t*-test was performed between engrafted-vehicle and engrafted-PQ groups for Figure 7C. Statistical significance of mice survival in Figure 7D was assessed by log-rank (Mantel-Cox) test. *p* values smaller than 0.05 were considered statistically significant.

## 3. Results

### 3.1. Identification of a New Drug Specifically Active on PEL Cells Using Phenotypic Screening Assay

**Figure 1:**
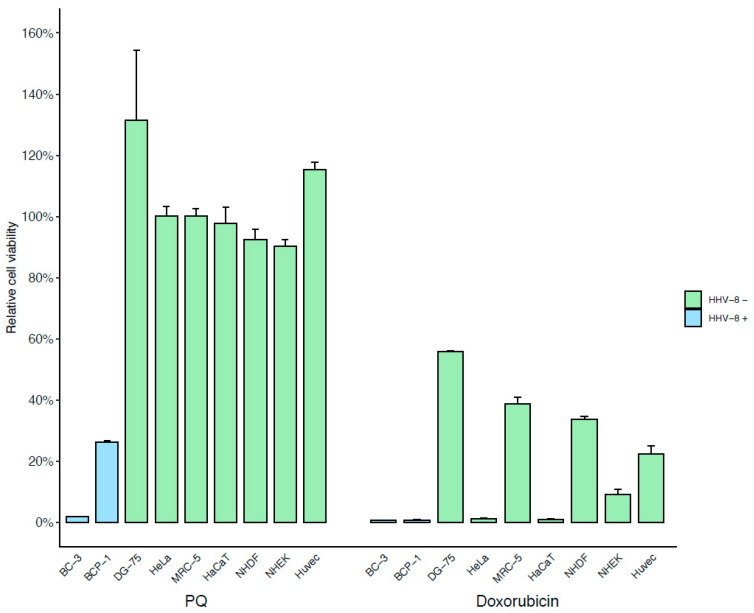
Specific cytotoxic effects of PQ treatment on PEL cell lines. HHV-8-infected PEL cell lines (BC-3, BCP-1) and multiple HHV-8-uninfected cells (DG-75, HeLa, MCR-5, and HaCaT cell lines and primary NHDF, NHEK, and HUVEC cells) were incubated with 10µM of PQ or doxorubicin (as control) for 48 h. Cell viability was assessed by CellTiter-Glo 2.0 assay of at least duplicate cultures and expressed as mean relative to their respective untreated control cells. Error bars represent the S.E.M for at least two independent experiments.

In order to identify a new candidate molecule lead for HHV-8-associated diseases, the Prestwick Chemical Compounds Library was examined by quantification of cell viability and apoptosis in an in vitro PEL (BC-3) cell line-based model. The antimalarial primaquine diphosphate (PQ) was selected from 2781 candidates based on its ability to induce cytotoxicity in more than 95% of BC-3 cells after 48 h of treatment and through apoptosis by a >4-fold increase in induction of caspase-3 and -7 activity after 24 h of treatment at 10 µM. To confirm the selectivity of PQ treatment to HHV-8-positive PEL cell lines, we tested the BCP-1 HHV-8-infected PEL cell line as well as several HHV-8-uninfected cells. We revealed that 10 µM PQ remarkably decreased the cell viability of the PEL cell lines, BC-3 and BCP-1. In contrast, at this concentration, PQ treatment did not affect HHV-8-uninfected cell viability (<10% of cell mortality), including the Burkitt’s lymphoma DG-75 cell line. In parallel, the chemotherapy doxorubicin, widely used for the treatment of KS and PEL [1,2,3,15], was used as a positive control of cell cytotoxicity. It is important to emphasize that, compared to PQ, doxorubicin induced a strong, non-specific cytotoxicity independently of the cell type (Figure 1).

The CC_50_ values of PQ in different cells are shown in Table 1 and were very low (average 5.1 ± 0.2 µM) in the HHV-8-infected cells while they were up to 65 times higher in the HHV-8-uninfected cells. Moreover, the cytotoxicity of PQ on BC-3 cells was also investigated and confirmed with a trypan blue exclusion assay and showed a decrease in cell viability by 73% and 96% at 10 and 20 µM, respectively, after 48 h of treatment. Furthermore, it appears that PQ-induced cytotoxicity was dose-dependent and began after 24 h of treatment (Supplementary Figure S1).

**Figure 2:**
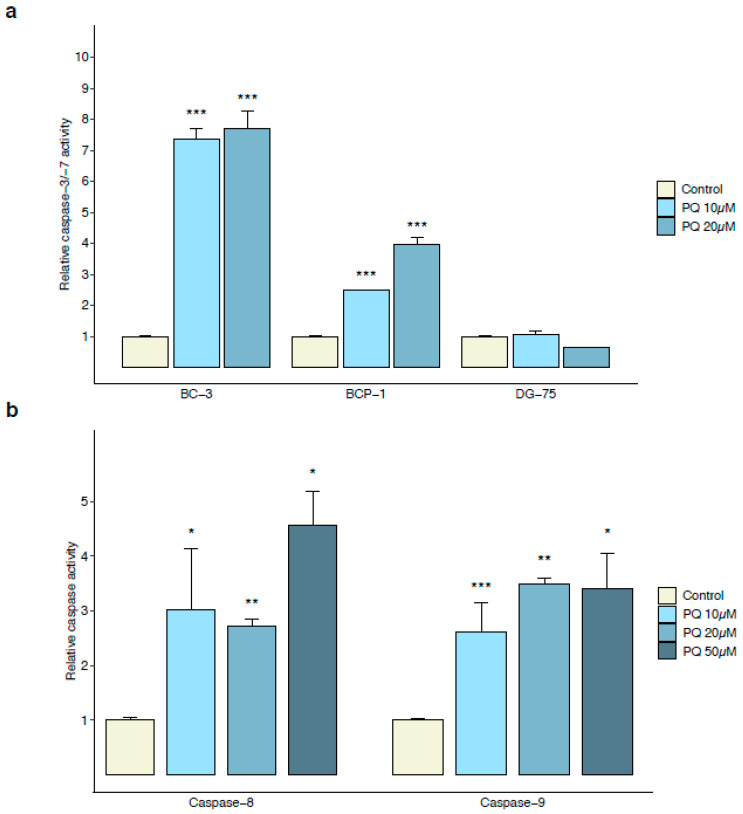
PQ induced caspase-dependent apoptosis in PEL cell lines. (**a**) Specific activation of the executioner caspase-3 and -7 in PQ-treated HHV-8-infected PEL cells. PEL cells (BC-3 and BCP-1) and HHV-8-uninfected Burkitt’s lymphoma cells (DG-75) were treated with PQ (10 and 20 µM) for 24 h followed by measurement of active caspase-3/-7 using the Caspase-Glo-3/-7 luminescent assay. Values are expressed as mean normalized to their respective untreated control cells. Error bars represent the S.E.M of triplicates for at least 3 independent experiments. ***, *p <* 0.001 vs. untreated control (Welch’s *t*-test). (**b**) Activation of both caspase-8 and -9 in PEL cells. BC-3 cells were incubated with increasing concentrations of PQ (0, 10, 20, and 50 µM) for 24 h and caspase-8 and -9 activity was assessed using Caspase-Glo-8 or -9 luminescent assays, respectively. Values are expressed as mean normalized to the untreated control cells. Error bars represent the S.E.M of triplicates. *, *p <* 0.05; **, *p <* 0.01; ***, *p <* 0.001 vs. untreated control (Wilcoxon–Mann–Whitney’s test).

To confirm the specific apoptotic-induced mechanism of PQ on PEL cells, we assessed caspase-3/-7 activity on BCP-1 and DG-75 cell lines and showed that PQ increased the caspase-3/-7 activity in HHV-8-infected BCP-1 and BC-3 cell lines but not in HHV-8-uninfected DG-75 cell line after 24 h of treatment (Figure 2a). Further analysis of PQ-induced apoptosis showed that 24 h of PQ treatment resulted in activation of caspase-8 and caspase-9 in the BC-3 cell line (Figure 2b). Thus, we identified the antimalarial drug PQ as a potent specific cytotoxicity inducer in HHV-8-infected PEL cells through the activation of both the intrinsic and extrinsic caspase-associated apoptotic pathways.

### 3.2. Primaquine Treatment Induces Oxidative Stress-Mediated Apoptosis in PEL Cells

**Figure 3:**
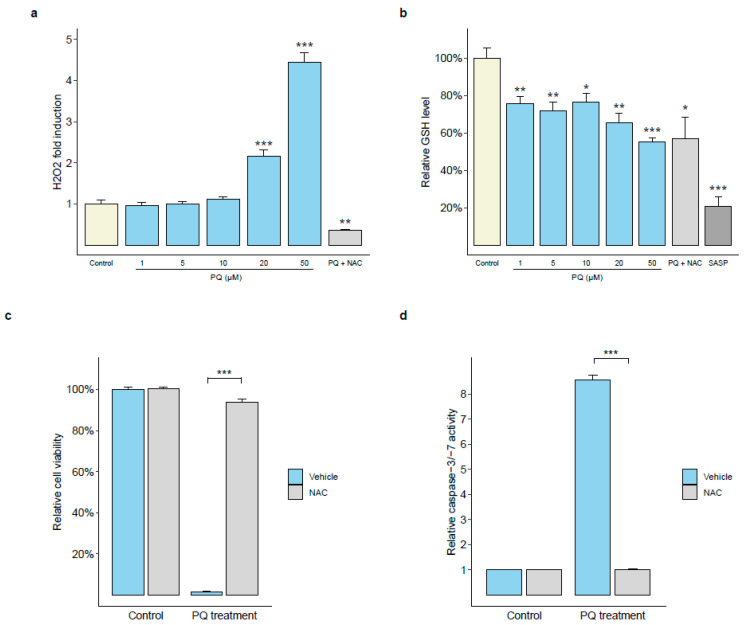
PQ induced cytotoxicity and apoptosis through ROS-dependent manner in PEL cell line. (**a**,**b**) PQ treatment induced oxidative stress in PEL cell line. BC-3 cells were incubated with increasing concentrations of PQ (1, 5, 10, 20, and 50 µM) for 24 h, then H_2_O_2_ and GSH were quantified using H2-DCFDA and monochlorobimane, respectively, and spectrofluorimetry. Some cells were pre-treated with 3 mM antioxidant *N*-acetylcysteine (NAC) for 12 h before incubation with 10 µM PQ. In total, 0.25 mM sulfasalazine (SASP), as a reducer of intracellular GSH levels, was used as a positive control. The GSH and H_2_O_2_ levels of untreated cells were defined as 100% (control) and as 1.0 (control), respectively. Values are expressed as mean normalized to H_2_O_2_ and GSH levels for the untreated control cells, respectively. Error bars represent the S.E.M of at least triplicates. *, *p <* 0.05; **, *p <* 0.01; ***, *p <* 0.001 vs. untreated control (Welch’s *t*-test). (**c**,**d**) The protective effect of antioxidant NAC on cytotoxic and apoptotic effects of PQ. BC-3 cells were pre-treated with (NAC) or without (vehicle) 3 mM NAC for 12 h before treatment with 10 µM PQ. Cell viability was assessed after 48 h of PQ treatment. The viability of PQ-untreated cells was defined as 100% (control). Caspase-3/-7 activity was evaluated after 24 h of PQ treatment. The caspase activity in PQ-untreated cells was defined as 1.0 relative light unit (control). Values are expressed as mean relative to the control. Error bars represent the S.E.M of triplicate cultures for three independent experiments. ***, *p <* 0.001 vs. PQ-treated vehicle group (Welch’s *t*-test).

It has previously been shown that cell death in PEL cells could be linked to oxidative stress through H_2_O_2_ production, which was prevented with pre-treatment with the antioxidant *N*-acetylcysteine (NAC) [16]. We found that PQ treatment in BC-3 cells induced the production of H_2_O_2_ at 24 h in a dose-dependent manner. In addition, at 24 h, we observed a progressive depletion of GSH levels as the levels of H_2_O_2_ and doses of PQ increased. Importantly, when pre-treating BC-3 cells with the antioxidant NAC, we were able to abolish the PQ-induced H_2_O_2_ production to levels lower than observed in untreated cells but failed to restore the GSH levels (Figure 3a,b).

We then sought to determine whether oxidative stress was related to the cytotoxicity and apoptosis activities induced by PQ. We found that the NAC pre-treatment was able to restore PQ-treated BC-3 cell viability as well as prevent the increase in caspase-3 and -7 activity induced by PQ (Figure 3c,d). These results strongly suggest that the ability of PQ to induce cytotoxicity in the PEL cell line is mediated in part by an increase in ROS production, leading to apoptosis through the activation of the caspases pathway. Therefore, oxidative stress appears to be a major process in the PQ mechanism of action on PEL cells.

### 3.3. Early Primaquine-Induced Apoptosis in PEL Cells Is Not Associated with Extracellular HHV-8 Virion Production

It has previously been reported that oxidative stress may induce latent HHV-8 reactivation or cell death in PEL cells in vitro [5,16]. As we had found that PQ treatment caused oxidative stress-mediated cell death, we sought to evaluate HHV-8 production in PQ-treated BC-3 cells.

**Figure 4:**
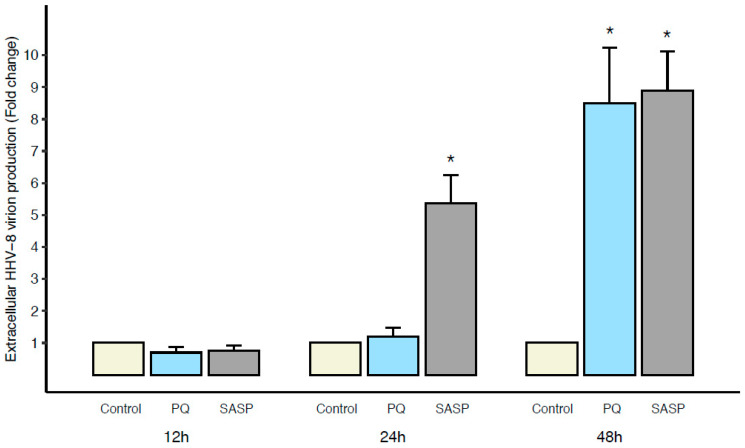
Extracellular HHV-8 virion production from PEL cells occurred after apoptosis induction by PQ. BC-3 cells were treated by PQ (10 µM) or SASP (0.25 mM as positive control) for 12 h, 24 h, or 48 h; then, extracellular HHV-8 virions were collected in the supernatant, as described in *Materials and Methods*. HHV-8 latency *LANA* DNA from each group was quantified by qPCR. The values of untreated control cells are defined as 1.0. Data are expressed as mean relative to their respective control at each individual time point (fold change). Error bars represent the S.E.M of triplicates for at least 2 independent experiments. *, *p <* 0.05 vs. untreated control (Wilcoxon–Mann–Whitney test).

BC-3 cells were treated with PQ for 12 h, 24 h, and 48 h and HHV-8 production in the supernatant was estimated by qPCR for the ORF-73 (*LANA*) gene. Sulfalazaline (SASP), which has been found to induce PEL apoptosis through oxidative stress and viral lytic gene expression and HHV-8 virion production, was used as a positive control in our experiments [17]. Compared to the untreated BC-3 cell supernatant, no virion production occurred after 12 h and 24 h of PQ treatment. However, after 48 h of PQ treatment, the HHV-8 virion production increased in the BC-3 cell supernatant (Figure 4). In comparison, the treatment of BC-3 cells with SASP increased virion production within as soon as 24 h while PQ did not. Moreover, it should be noted that at 24 h of PQ treatment, BC-3 cell viability had already decreased by 50% (Supplementary Figure S1) and caspases’ activity was increased (Figure 2a,b). Therefore, these data strongly suggest that the induction of cell death by PQ occurred independently of viral lytic replication but also that the persistence of PQ treatment and increasing oxidative stress could lead to a transient increase in HHV-8 virion production.

### 3.4. Four Cellular Genes Involved in Apoptotic Process Are Up-Regulated during Primaquine Treatment of HHV-8-Infected Cells

In order to determine the genes and pathways involved in the PQ-induced apoptosis in HHV-8-infected cells, we carried out RNA-sequencing analysis to assess the variations in the transcriptional profiles of both cellular and viral genes following 12 h of PQ treatment.

**Figure 5:**
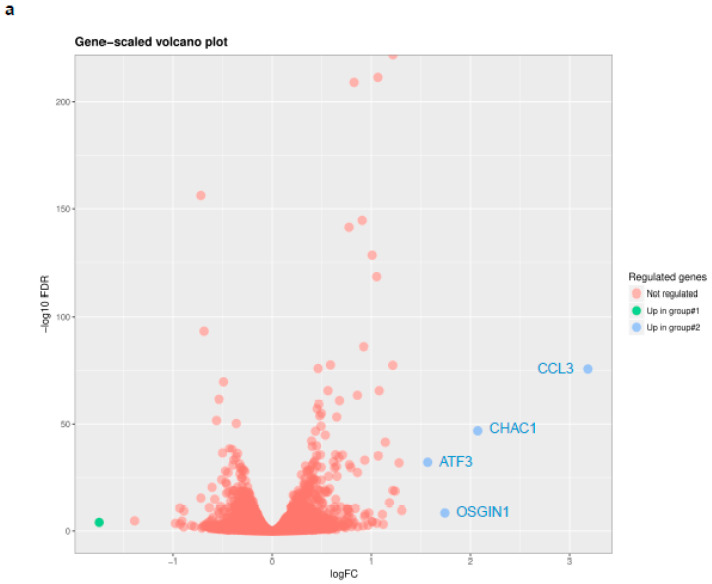

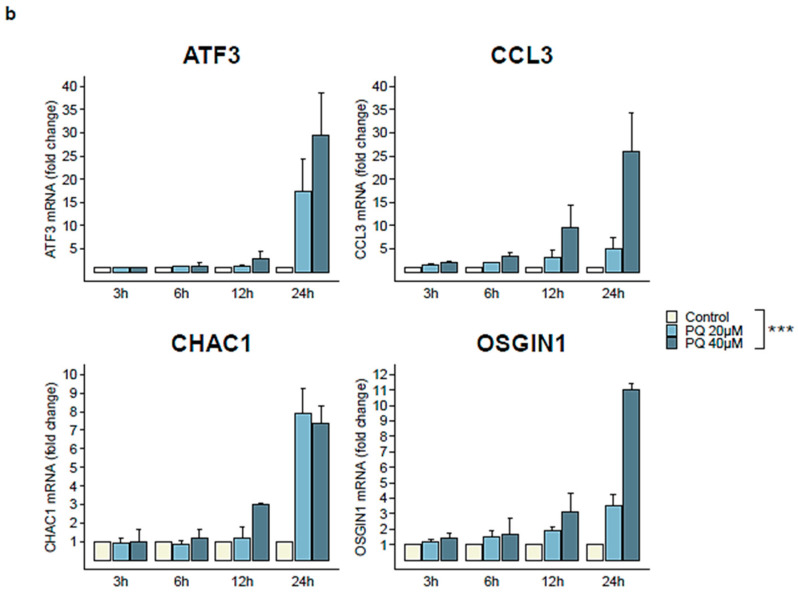
Four main cellular candidate genes were up-regulated by PQ treatment in PEL cell line. (**a**) RNA-seq analysis of cellular genes differentially expressed between PQ-treated and untreated BC-3 cells. RNA-seq was performed on the triplicate PQ-treated or untreated BC-3 cell lines, and differentially expressed cellular genes were detected within PQ-treated BC-3 cells when compared with untreated control. The cut-off values selected for the analysis of the data were: *p*-value threshold, 0.05; log2foldchange threshold, 1.5; count per million (CPM) threshold, 1. (**b**) Relative expression of the four main candidate genes by qRT-PCR. BC-3 cells were incubated for 3–24 h with PQ (20 µM and 40 µM). Total RNA was extracted, and *OSGIN1*, *ATF3*, *CHAC1,* and *CCL3* mRNA expression were determined by qRT-PCR and normalized to β-actin in triplicate samples. The values of the untreated control are defined as 1.0. Data are expressed as mean relative to their respective untreated control at each individual time point (fold change). Error bars represent the SD for two independent experiments. ***, *p <* 0.001 vs. untreated control (two-way ANOVA).

Using RNA-sequencing analysis, we showed that four cellular transcripts were up-regulated after PQ treatment and corresponded to *OSGIN1* (oxidative stress-induced growth inhibitor 1), *ATF3* (activating transcription factor 3), *CHAC1* (cation transport regulator-like 1), and *CCL3* (MIP-1-α macrophage inflammatory protein1-α) genes (Figure 5a and Table 2). Importantly, RNA-seq analysis also revealed that PQ did not reduce the transcriptional expression of the HHV-8 latent genes, *v-cyclin*, *LANA,* and *v-FLIP*, or increase the transcriptional expression of the lytic genes, such as *Rta* (Table 2). These results indicate that PQ has no direct effect on viral gene expression after 12 h of treatment.

Overall, pathway enrichment analysis showed that, as cellular biological functions, only “programmed cell death” and “apoptosis” were affected within PQ-treated BC-3 cells (Supplementary Figure S2).

We then assessed the four genes *CHAC1*, *OSGIN1*, *ATF3,* and *CCL3* for validation of their transcriptional change by qRT-PCR. Our results confirmed the up-regulation of these genes after 12 h PQ treatment and showed that 24 h of 20 µM PQ treatment was able to increase the *OSGIN1*, *CCL3*, *CHAC1,* and *ATF3* expressions by 3.5-fold, 4.9-fold, 8-fold, and 17.3-fold, respectively, when compared with untreated cells (Figure 5b).

### 3.5. Primaquine Treatment Induces ER Stress-Mediated Apoptosis through Transcriptional Expression of CHOP and Activation of Caspase-4

Environmental and genetic factors that disrupt endoplasmic reticulum (ER) function may cause an accumulation of misfolded and unfolded proteins in the ER lumen, a condition termed ER stress. ER stress activates a signaling network called the Unfolded Protein Response (UPR), which causes a decrease in protein synthesis while preventing the aggregation of unfolded proteins to alleviate this stress and restore ER homeostasis, promoting cell survival and adaptation [18,19,20]. However, under persistent and/or irremediable ER stress, the UPR signaling pathway switches from pro-survival to pro-apoptotic. ER stress-mediated apoptosis is regulated, in part, by the transcriptional induction of C/EBP homologous protein (CHOP, also named growth arrest, and DNA-damage-inducible 153, GADD153), and the activation of caspase-4 (an ER outer membrane-localized caspase only activated by ER stress) [21,22,23].

**Figure 6:**
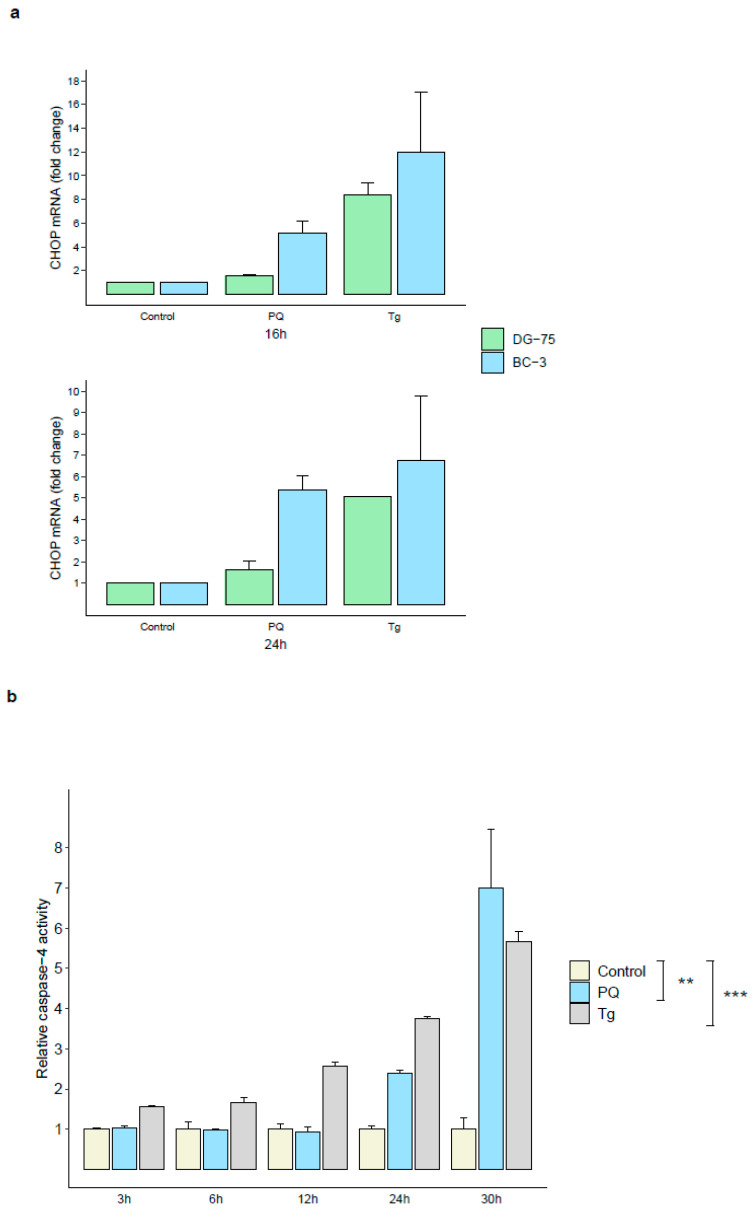
PQ treatment induced ER stress-mediated apoptosis in PEL cells. (**a**) Increase in CHOP mRNA level in 16 h and 24 h PQ treated-BC-3, but not in DG-75 cell lines. HHV-8-infected BC-3 cell line and HHV-8-uninfected DG-75 cell line were treated with PQ (20 µM) or Tg (3 µM as positive control) for 3–24 h. *CHOP* mRNA expression was determined by qRT-PCR and normalized to β-actin in triplicate samples. The values of the untreated control are defined as 1.0. Data are expressed as mean relative to their respective untreated control at each individual time point (fold change). Error bars represent the S.E.M for two independent experiments. (**b**) PQ induced ER-associated caspase-4 activation in BC-3 cell line. BC-3 cells were treated with PQ (10 µM) or Tg (1 µM as positive control) for 3–30 h followed by measurement of active caspase-4 using the fluorometric caspase-4 assay as described in *Materials and Methods*. The values of the untreated control are defined as 1.0. Data are expressed as mean relative to their respective untreated control at each individual time point (fold change). Error bars represent S.E.M for three independent experiments. **, *p <* 0.01; ***, *p <* 0.001 vs. untreated control (two-way ANOVA analysis and Dunnett’s multiple comparisons test).

We have previously shown that PQ treatment induced transcriptional up-regulation of *CHAC1* and *ATF3*, two genes known to be important components of the pro-apoptotic programs following UPR activation. We then investigated the effects of PQ on the pro-apoptotic *CHOP* expression and showed that it was up-regulated by 5.1-fold and 5.4-fold in BC-3 cells after 16 h and 24 h of PQ treatment, respectively. In parallel, we used the ER stress inducer thapsigargin (Tg) [21,24] as a positive control and showed an up-regulation of its expression as well. However, it is interesting to note that DG-75 cells showed no increase in *CHOP* expression under PQ treatment, indicating a specific effect of PQ on pro-apoptotic ER stress-inducible *CHOP* expression in HHV-8-infected cells (Figure 6a).

Moreover, after 24 h and 30 h of PQ treatment, we revealed an increase of 2.4-fold and 7-fold of caspase-4 activity in BC-3 cells, respectively. These results were similar to those obtained in Tg-treated BC-3 cells (Figure 6b).

Altogether, these results suggest ER stress-mediated apoptosis and pro-apoptotic UPR signaling as part of the specific apoptotic effects induced by PQ in PEL cells.

### 3.6. Primaquine Treatment Reduces PEL Tumor Growth and Improves Survival in a NOD/SCID Mice Xenografted with a PEL

**Figure 7:**
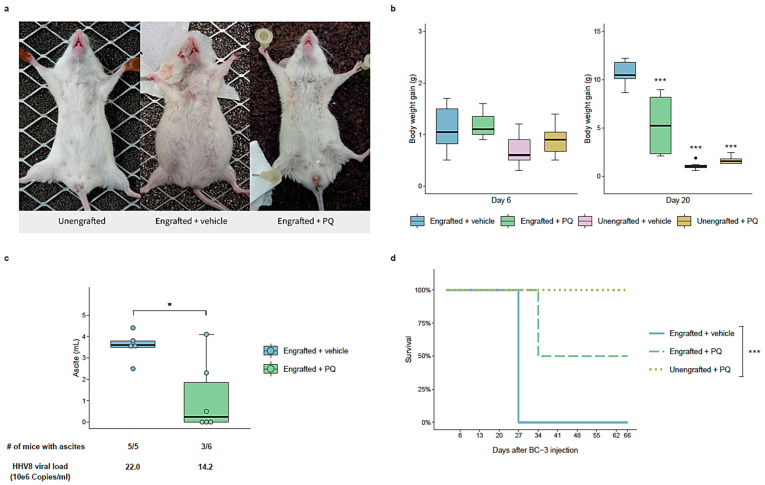
PQ reduced PEL progression in a NOD/SCID mice PEL model. In total, 10^7^ BC-3 cells (BC-3-engrafted) or PBS (BC-3-ungrafted) were injected intraperitoneally (i.p) in NOD/SCID mice (*n* = 6 per group). Beginning 24 h later, PQ (25 mg/kg) or PBS (vehicle) was administrated via i.p 3 days per week. The mice were euthanized when moribund or showing signs of discomfort and/or when weight reached 150% of their initial weight. (**a**,**c**) Representative photographs of animals and ascites fluid volumes were collected immediately postmortem. (**b**) The body weight of each mouse was recorded every week. Median body weight gain of mice is represented for each indicated group with a box plot 6 days and 20 days after BC-3 cell injection. ***, *p <* 0.001 vs. engrafted + vehicle group (two-way ANOVA analysis and Tukey’s multiple comparisons test). (**c**) Distribution of tumor ascites volume is represented with a box plot within engrafted + PQ and engrafted + vehicle groups. Ascites incidence and HHV-8 viral load (HHV-8 copy number/mL) are also shown for each group. One of the mice of the engrafted vehicle-treated group was removed from this analysis because it showed severe alterations in its health and had to be sacrificed despite presenting no production of ascites. *, *p <* 0.05 vs. engrafted + vehicle group (Welch’s *t*-test). (**d**) Mice survival is represented with a Kaplan–Meier curve. ***, *p <* 0.001 log-rank (Mantel–Cox) test.

As PQ treatment causes significant and selective apoptosis of the HHV-8-infected PEL cell lines, we sought to evaluate its pro-apoptotic activity against HHV-8-associated tumor growth in a PEL xenograft in vivo model in NOD/SCID mice, as previously described [5,25,26]. Two separate experiments were performed in order to assess the effect of two concentrations of PQ at 12.5 mg/kg and 25 mg/kg by intraperitoneal injection. HHV-8-infected BC-3 cells intraperitoneally injected into the peritoneal cavity of mice resulted in a rapid increase in body weight associated with massive ascites with abdominal distention within 2 weeks (Figure 7 and Supplementary Figure S3). The engraftment of mice with BC-3 cells was confirmed by qPCR analysis for the presence of HHV-8 and albumin from peritoneal solid samples of sacrificed mice at the end of the experiment, representing 3.47 × 10^7^ and 4.08 × 10^7^ median copy per 10^6^ BC-3 cells for the vehicle engrafted and PQ-treated engrafted mice, respectively.

We revealed a reduction of 28% in the body weight of engrafted mice at day 24 with the administration of the lower dose of PQ (12.5 mg/kg), and a decrease in the median ascites volume of 24% (Supplementary Figure S3A,B). At the higher dose of PQ (25 mg/kg), we observed a reduction of 49% in the median body weight of engrafted mice after 20 days (Figure 7b) and a decrease of 93% in the median ascites volume compared with vehicle group (Figure 7c), consistent with the difference of abdominal distension observed in Figure 7a. Importantly, three out of six mice of the PQ-treated group showed no apparent increase in body weight until the end of the study, with no formation of malignant ascites (Figure 7b–d) and no signs of pain nor distress observed. These data demonstrated a significant delay in tumor formation in PQ-treated mice. We also observed a decrease in the HHV-8 viral load in ascites in PQ-treated mice in a dose-dependent manner, shown by a decrease of 10.6% and 35.4% HHV-8 copy number/mL in 12.5 mg/kg and 25 mg/kg PQ-treated mice, respectively (Figure 7c and Supplementary Figure S3B). In addition, the survival time was improved from 27 days in PBS-injected engrafted mice to 66 days (the end of the study) in half of the 25 mg/kg PQ-treated engrafted mice group (Figure 7d). Importantly, the two doses of PQ were well tolerated in mice without any overt toxic effects (Figure 7b,d and Supplementary Figure S3A).

### 3.7. Primaquine Treatment Decreases Kaposi’s Sarcoma-Related Lesions and Lymphedema in Humans

**Figure 8:**
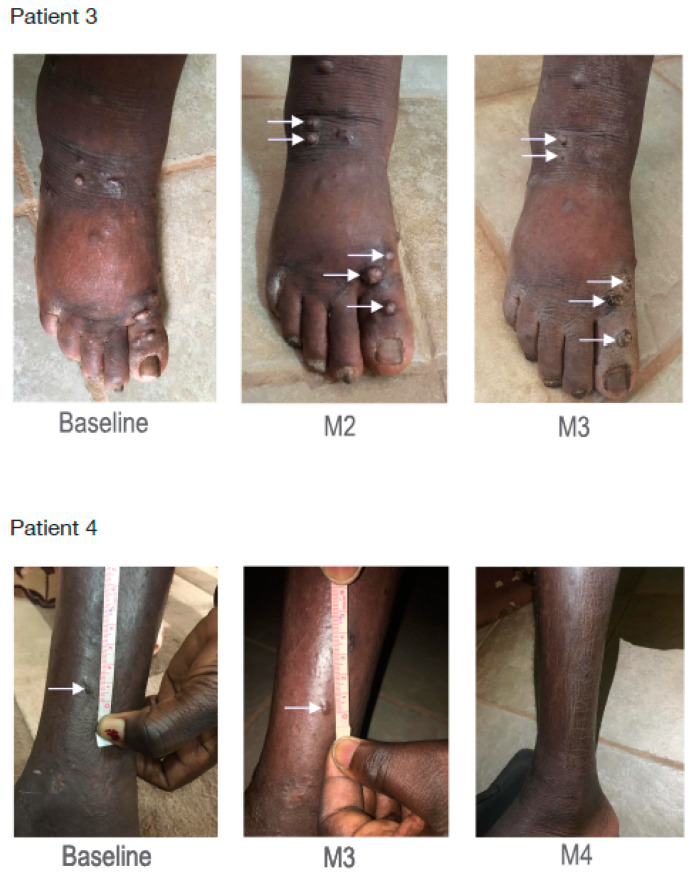
PQ reduced KS-related lesions and lymphedema. Clinical effects in patients 3 and 4: white arrows indicated KS lesions with clinically proven regression. These pictures also evidenced lymphedema improvement in both patients.

We then investigated the effect of PQ in humans harboring severe Kaposi’s sarcoma in a proof-of-concept pilot trial with the main objectives being to assess the efficacy, the safety, and the tolerability of PQ. P1 (patient 1) presented a clinical progression between baseline and M2 with an occurrence of 18 new KS lesions, a complete disappearance of two KS lesions, and an improvement in the lymphedema of the right leg. The treatment was stopped and chemotherapy with paclitaxel was started in order to achieve a good clinical improvement. P2 had at M3 an improvement in five out of the eight KS lesions present at baseline. He did not harbor any lymphedema at any time of the trial. P3 harbored at M3 an improvement in five out of the six KS lesions present at baseline on the right foot and an improvement in lymphedema between baseline and M3. P4 harbored at M3 a complete disappearance of three out the four KS lesions present at baseline and an improvement in lymphedema of the right leg. The clinical investigator decided to pursue the duration of treatment up to M4, and all the KS lesions and the lymphedema totally disappeared (Figure 8 and Supplementary Table S2). Importantly, primaquine was well tolerated and no adverse event was observed in patients treated with primaquine.

## 4. Discussion

As the current treatment for HHV-8-associated diseases has limited efficacy, the identification of a more specific therapeutic approach remains an unmet medical need. In this study, through a comprehensive drug screening of over 2700 candidates, we identified for the first time the antimalarial primaquine diphosphate as a promising targeted therapeutic agent to treat HHV-8-associated PEL and KS. We demonstrated in vitro that primaquine is specific to HHV-8-infected PEL cell lines compared to HHV-8-uninfected cells and induces cytotoxic effects through executioner caspases-3/-7-dependent apoptosis. Deciphering the apoptotic process induced by primaquine, we demonstrated the activation of caspase-8 and -9, indicating the involvement of both intrinsic and extrinsic apoptosis pathways in primaquine-induced cell death in PEL cells.

ROS and oxidative stress are known as apoptotic triggers and modulators of cell death [27,28]. As primaquine-induced oxidative stress has been closely related to the hemolytic toxicity of primaquine and some of its metabolites in G6PD-deficient erythrocytes [29,30], we sought to evaluate its pro-oxidant effect in PEL cells. Interestingly, primaquine promoted intracellular ROS generation associated with GSH depletion. Pre-treatment with the antioxidant *N*-acetylcysteine dramatically reduced ROS generation and strongly relieved primaquine-induced cytotoxicity and caspases-3/-7 activation. These results indicated that ROS generation has a major role in the primaquine apoptotic process in PEL cells. This finding was supported by the up-regulation of the expression of *OSGIN1* observed in primaquine-treated PEL cells, a tumor suppressor gene induced by oxidative stress that has been previously reported to play a role in PEL cells apoptosis, in part through the regulation of ROS production and GSH synthesis [17].

Previous studies have reported that the levels of ROS are critical to regulating the balance between HHV-8 reactivation and PEL cell death as high levels of ROS led mostly to cell death [16]. We observed that prolonged incubation of 48 h of BC-3 cells with primaquine was associated with increased extracellular HHV-8 virion production. As massive cell death was induced by primaquine within 48 h, it would be logical for the virus to sense the cell death and stress signals leading to an escape from those dying cells. However, it is not fully elucidated whether these virions came from the viral reactivation of latent cells or from increased release of already formed HHV-8 virions in lytic PEL cells. Importantly, our data did not show any direct influence of primaquine on the expression of latent and lytic viral genes as well as on virion production until 24 h of treatment, while apoptotic mechanisms have already been activated that lead to PEL cell death (Supplementary Figure S1). Moreover, we did not observe any increase in HHV-8 viral load in ascites or in the intraperitoneal solid sample from NOD/SCID PEL xenograft mice treated with primaquine. These findings strongly indicate that the induction of cell death by primaquine occurred independently of viral lytic replication.

As we further explored the cell death signaling pathways involved in primaquine mechanisms of action, our RNA-seq analysis highlighted the activation of some hallmarks of the ER stress-mediated apoptosis signaling pathway. Due to their enhanced rate of growth and proliferation, the high protein demand of cancer cells can trigger ER stress and, subsequently, UPR to maintain ER homeostasis, and enables tumor cell survival [20,31]. However, if the ER stress is too severe, persistent, or cannot be resolved, the pro-apoptotic pathways of the UPR are activated, including the induction of the pro-apoptotic transcription factor CHOP and activation of caspase-4, leading to apoptosis [23,32,33,34,35]. Thus, ER stress-targeting therapy, by triggering pro-apoptotic pathways of the UPR, represents an interesting strategy for anti-tumor, including anti-PEL, therapeutics [19,20,21,36,37]. Remarkably, in addition to ER stress-dependent caspase-4 activation, our study showed the increased expression of *ATF3* and *CHAC1*, as well as a specific induction of *CHOP,* in primaquine-treated PEL cells. CHAC1 is a pro-apoptotic ER stress-inducible gene, downstream of ATF3 and CHOP, which can mediate, in part, the pro-apoptotic effects of both these transcription factors [22,38,39]. Further analysis would be necessary to gain insight into the molecular mechanism of the activation of the ER stress-mediated apoptosis signaling. However, our data can evoke the activation of the pro-apoptotic ER stress-inducible gene to be involved in the apoptosis caused by primaquine in HHV-8-infected cells.

Finally, it has been reported that patterns of HHV-8 expression and the mechanisms associated with viral latency are very similar in every infected cell [9,40], contributing to HHV-8-induced malignancies and pathogenesis. Hence, we hypothesize that a molecule targeting signaling pathways associated with the cell death of latently infected cells would eradicate different latently infected cell types, leading to the curing of HHV-8-associated malignancies. Supported by our in vitro and in vivo primaquine anti-tumor efficacy and safety findings in PEL cells and mouse models, and also by the fact that primaquine has been used worldwide since the 1950s with remarkable tolerance among glucose 6-phosphate dehydrogenase (G6PD)-normal patients [41], an exploratory proof of concept clinical trial on Kaposi’s sarcoma was conducted. This pilot clinical study demonstrated some positive anti-tumor efficacy of primaquine on Kaposi’s sarcoma-related lesions and lymphedema and was well tolerated without any adverse events. These results encourage extending to a larger clinical trial of KS and/or PEL patients treated with dose ranges of primaquine to better evaluate its therapeutic efficacy. Importantly, a robust G6PD deficiency diagnostic prior to offering primaquine therapy would greatly reduce the risk of drug-induced acute haemolytic anaemia in vulnerable individuals.

## 5. Conclusions

In conclusion, the antimalarial primaquine diphosphate induces cell death through apoptosis induction, specifically in HHV-8-infected PEL cells, involving oxidative stress- and ER stress-mediated apoptosis pathways. The dose-dependent, anti-tumor efficacy of primaquine in vivo in a PEL mouse model and in patients harboring severe KS as well as its good tolerance without significant side effects suggest that the effects of primaquine are specific to HHV-8-infected tumor cells, thereby raising the possibility that primaquine may serve as a novel, promising targeted therapeutic agent in the treatment of, at least, HHV-8-associated PEL and KS.

## Figures and Tables

**Table 1:** The CC50 values of PQ in HHV-8-infected and HHV-8-uninfected cells. CC50, cytotoxic concentration that reduces cell viability by 50% after 48 h of PQ treatment. The CC50 values were calculated using GraphPad Prism 6.0.

Cell Line	BC-3	BCP-1	DG-75	HaCaT	HeLa P4	MRC-5	NHDF	NHEK	HUVEC
**PQ (µM)**	4.93	5.33	80.51	47.37	90.2	89.02	98.05	40.74	331.1

**Table 2:** RNA-seq analysis of the main differentially modulated cellular and viral genes from PQ-treated BC-3 cells. RNA-seq analysis of transcriptional expression regulation of the four main cellular genes, ATF3, CCL3, CHAC1, and OSGIN1, and of the three main latent viral genes, LANA, v-FLIP, and v-cyclin and the main lytic viral gene Rta in PQ-treated BC-3 cells compared to untreated control. Their type, log of fold change, and status of regulation in PQ-treated group compared to untreated control group are shown. ATF3, activating transcription factor; CCL3, MIP-1-α, macrophage inflammatory protein1-α; CHAC1, cation transport regulator-like 1; OSGIN1, oxidative stress-induced growth inhibitor 1; v-FLIP, viral Fas-associated protein with death domain-like interleukin-1β-converting enzyme/caspase-8-inhibitory protein; v-cyclin, viral cyclin; LANA, Latency-Associated Nuclear Antigen; RTA, Replication and Transcription Activator.

Gene	Type	logFC	Status in PQ-Treated Group
ATF3	Cellular	1.57	Up-regulated
CCL3	Cellular	3.18	Up-regulated
CHAC1	Cellular	2.07	Up-regulated
OSGIN1	Cellular	1.74	Up-regulated
v-FLIP	Viral (latent)	−0.15	Not regulated
v-Cyclin	Viral (latent)	−0.2	Not regulated
LANA	Viral (latent)	0.22	Not regulated
RTA	Viral (lytic)	0.1	Not regulated

## Data Availability

The data presented in this study are available on request from the corresponding author.

## Supplementary Materials

The following supporting information can be downloaded at: https://www.mdpi.com/article/10.3390/cancers14030543/s1. Figure S1. Kinetic of cytotoxic effects of PQ on BC-3 cell line, Figure S2. Pathway enrichment analysis from RNA-sequencing analysis, Figure S3. PQ reduced PEL progression in a NOD/SCID mice PEL model, Table S1. List of primers used for qRT-PCR, Table S2. Summary of clinical trial results from a proof-of-concept trial in human harboring severe Kaposi’s sarcoma.
