# Supplementary material for: Primaquine as a Candidate for HHV-8-Associated Primary Effusion Lymphoma and Kaposi’s Sarcoma Treatment
Source: Cancers (Basel). 2022 Jan 21;14(3):543. doi: 10.3390/cancers14030543 (PMC8833810; doi:10.3390/cancers14030543)
Supplement: Supplementary file 1 [file cancers-14-00543-s001.zip › cancers-1536846-supplementary.pdf]

cancers

MDPI

Supplementary Material

Primaquine as a candidate for HHV-8-associated primary effusion lymphoma and Kaposi's sarcoma treatment

Adélie Gothland [1,*], Valentin Leducq [1], Philippe Grange [2,3], Ousmane Faye [4], Laurianne Beauvais Remigereau [1], Sophie Sayon [1], Nathalie Désiré [5], Aude Jary [1], Emmanuel Laplantine [6], Almoustapha Issiaka Maiga [7,8], Nicolas Dupin [2,3], Anne-Geneviève Marcelin [1] and Vincent Calvez [1]

Service de Virologie, Hôpital Pitié Salpêtrière, Institut Pierre Louis d'Epidémiologie et de Santé Publique (iPLESP), INSERM UMR_1136, Sorbonne Université, 75013 Paris, France ; valentin.leducq@sorbonne-universite.fr (V.L.); lauriannebr@lbrconseil.fr (L.B.R.); sophie.sayon@aphp.fr (S.S.); aude.jary@aphp.fr (A.J.); anne-genevieve.marcelin@aphp.fr (A.-G.M.); vincent.calvez@aphp.fr (V.C.)
2 Cutaneous Biology Lab, INSERM U1016, UMR8104, Institut Cochin, Université de Paris, 24 rue du Faubourg St Jacques, 75014 Paris, France; philippe.grange@aphp.fr (P.G.); nicolas.dupin@aphp.fr (N.D.)
3 Service de Dermatologie, CeGGID et CNR IST Bactériennes, Hôpital Cochin site Port Royale, AP-HP, Groupe Hospitalier Paris Centre Cochin-Hôtel Dieu-Broca, 123 boulevard de Port Royal, 75014 Paris, France
4 Département de Dermatologie, Faculté de Médecine et de Pharmacie, Université de Bamako, BP 1805 Bamako, Mali; faye_o@yahoo.fr
5 Institut Pierre Louis d'Epidémiologie et de Santé Publique, INSERM, Assistance Publique—Hôpitaux de Paris, Sorbonne Université, 75012 Paris, France; nathalie.desire@sorbonne-universite.fr
6 Center for Immunology and Microbial Infections—CIMI-Paris, Sorbonne Université, INSERM, CNRS, 75013 Paris, France; emmanuel.laplantine@upmc.fr
7 Unité d'Epidémiologie Moléculaire de la Résistance du VIH aux ARV, SEREFO, FMOS, University of Sciences, Techniques and Technologies of Bamako, BP 1805 Bamako, Mali; almoustapha@gmail.com
8 Clinical and Microbiology Laboratory, University Hospital Gabriel Toure, BP 1805 Bamako, Mali
* Correspondence: adelie.gothland@gmail.com; Tel.: +33-1-42-17-58-44

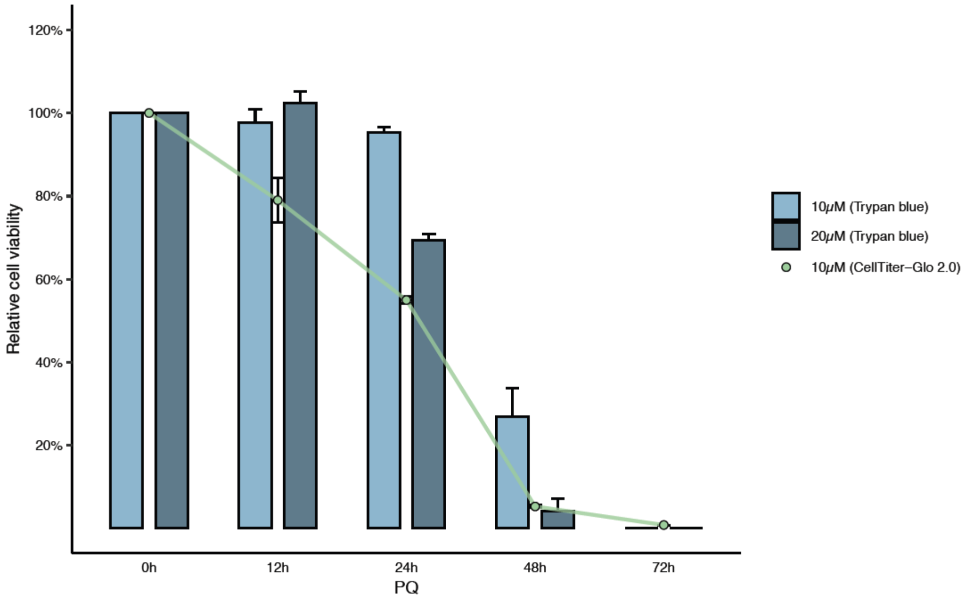

Figure S1. Kinetic of cytotoxic effects of PQ on BC-3 cell line. HHV-8-infected PEL cell lines, BC-3, were incubated with 10µM or 20µM of PQ for 12h, 24h, 48h and 72h. Cell viability was assessed by CellTiter Glo 2.0 and trypan blue exclusion assays, expressed as mean relative to the untreated control cells. The number of viable cells in untreated cells on each time point was considered as 100%. Data represent S.E.M of triplicate cultures.

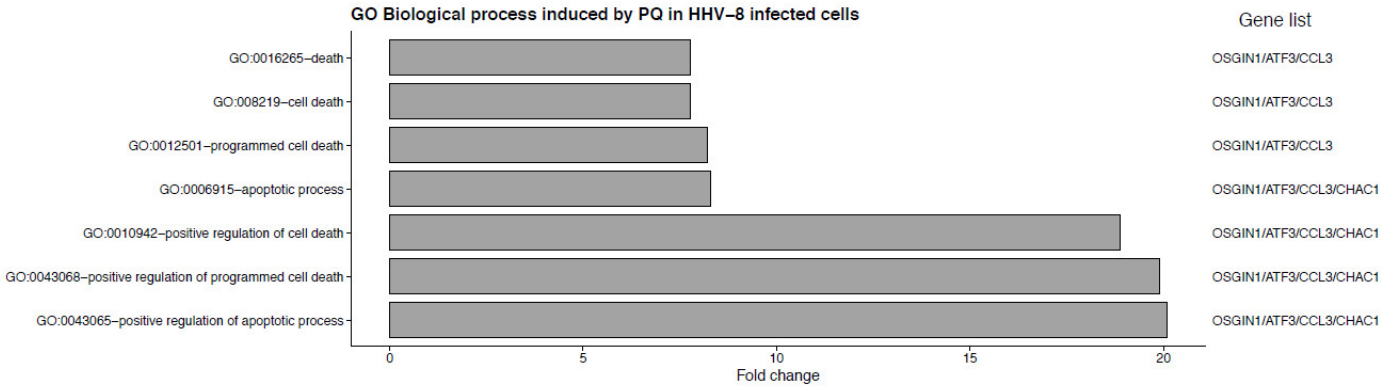

Figure S2. Pathway enrichment analysis from RNA-sequencing analysis. Pathway enrichment (Gene Ontology, GO) analysis of differentially modulated genes classified by their biological functions and arranged according to their fold change. Pathway enrichment analysis was conducted with Fisher test using R package (clusterProfiler). The 7 most significant ($P < 0.05$) biological process Gene Ontology (GO) terms, their fold change, and gene list are shown.

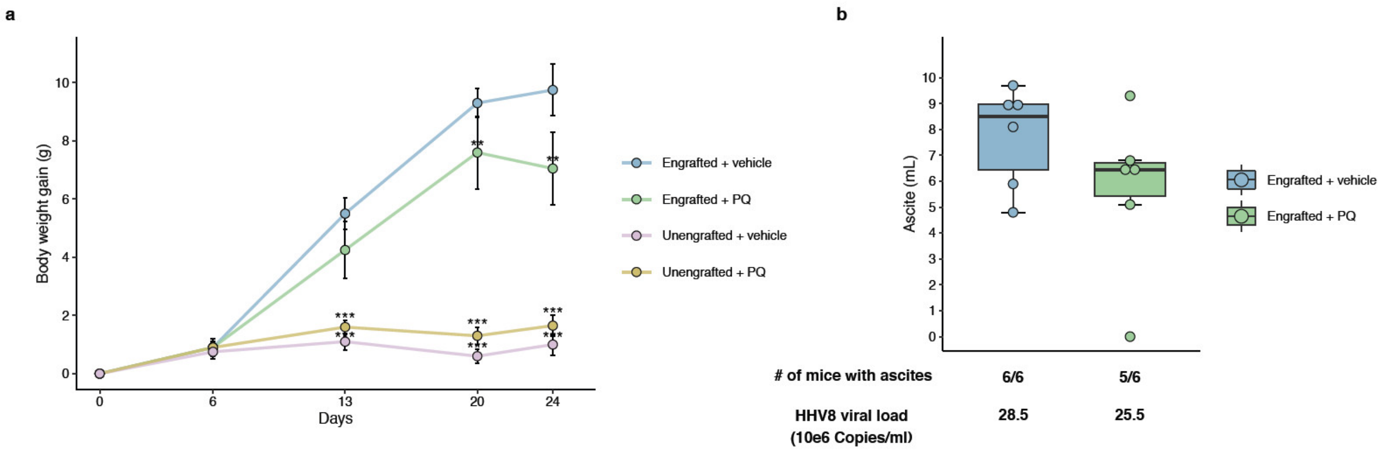

Figure S3. PQ reduced PEL progression in a NOD/SCID mice PEL model. 2.10^7 BC-3 cells (Engrafted) or PBS (Unengrafted) were injected intraperitoneally (i.p) in NOD/SCID mice ($n = 6$ per group). Beginning 24h later, PQ (12.5mg/kg) or PBS (vehicle) were administrated i.p 3 days per week. (**A**) The body weight of each mouse was recorded every week. Median body weight gain of mice are represented for each indicated group from 0 to 24 days after BC-3 cells injection. **, $P < 0.01$; ***, $P < 0.001$ (two-way ANOVA analysis and Tukey's multiple comparisons test). (**B**) Distribution of tumor ascites volume are represented with a box plot within engrafted + PQ and engrafted + vehicle groups. Ascites incidence and HHV-8 viral load (HHV-8 copy number/ml) are also shown for each group.

Table S1. List of primers used for qRT-PCR. qRT-PCR, real time quantitative reverse transcription-polymerase chain reaction; *OSGIN1*, oxidative stress-induced growth inhibitor 1; *ATF3*, activating transcription factor; *CHAC1*, cation transport regulator-like 1; *CCL3*, *MIP-1-α*, macrophage inflammatory protein1-α; *CHOP*, C/EBP homologous protein.

Gene	Sequences
OSGIN1	GCCTGGCACTCCATCGAA
	TTCGCTTCTTCTGCATCCAGT
ATF3	CAAAAGCCGAGGTAGCCCC
	CAGGCACTCCGTCTTCTCC
CHAC1	GCAGGGAGACACCTTCCATC
	GTATGCCACGCCCCAAGTG
CCL3	CATCACTTGCTGCTGACACG
	CTGGCTGCTCGTCTCAAAGT
CHOP	AGAACCAGGAAACGGAAACAGA
	TCTCCTTCATGCGCTGCTTT
β-actin	CCAGCTCACCATGGATGATG
	ATGCCGGAGCCGTTGTC

Table S2. Summary of clinical trial results from a proof-of-concept trial in human harboring severe Kaposi's sarcoma. Information collected from listed references or oral communication with the treating dermatologists. OD, once a day; BID, twice a day; KS, Kaposi's sarcoma; PQ, Primaquine diphosphate.

	Baseline	Month 2	Month 3	Month 4
**Patient #1. 15mg PQ/OD**	16 KS lesions	18 new KS lesions 2 disappearance Withdrawn		
	Lymphedema right and left legs	Clinical improvement		
**Patient #2. 15 mg PQ/OD**	8 KS lesions		5/8 KS lesions improved	
	No Lymphedema		No Lymphedema	
**Patient #3. 15 mg PQ/BID**	6 KS Lesions		5/6 KS lesions improved	
	Lymphedema right foot		Clinical improvement	
**Patient #4. 15 mg PQ/BID**	4 KS Lesions		3/4 KS Lesions disappearance	All lesions cured
	Lymphedema right leg and right foot		Clinical improvement	Complete disappearance
